# Detection of the Content of Two Coumarins, IM and ISOIM, and Their Mechanism of Action on Colitis Rats in Angelica albicans

**DOI:** 10.1155/2022/5475559

**Published:** 2022-07-16

**Authors:** Juan Zhang, Leilei Dong, Ying Pan

**Affiliations:** Department of Pharmacy, Third Hospital of Hebei Medical University, Shijiazhuang, 050000 Hebei, China

## Abstract

*Angelica albicans* is being used in the cure of different, respiratory, neuromuscular, and cutaneous diseases in traditional eastern medicine. The pharmacokinetic (PK) characteristics of imperatorin (IM) and isoimperatorin (ISOIM), the main effective components in *Angelica albicans*, were investigated. The rapid, subtle, and measuring the PKs of a drug, a validated UPLC/MS/MS methodology was designed for a total of 2 furanocoumarins in 2,4,6-trinitrobenzene sulfonic acid-stimulated and untreated mice. After that, blood samples were obtained. *Angelica albicans* (0.5 and 1.0 g/kg) was given orally, taken regularly from the tail vein. The time it takes for colitis rats to achieve their maximal concentration (*T*_max_) imperatorin and isoimperatorin was considerably postponed. In comparison to normal rats, all furanocoumarins had lesser peak plasma concentrations (*C*_max_) and higher represent residence durations. The area below the *C*_max_ time-curve or clearance half-life did not differ significantly. In normal rats, all two furanocoumarins attained maximal plasma levels between 40 and 75 minutes, demonstrating fast oral absorption. The periods to attain *T*_max_ of the two furanocoumarins, on the other hand, were shorter than in earlier studies. Therefore, colitis-linked alterations in the drug-absorption stage may result in a late *T*_max_ and lowered *C*_max_, which have no effect on its clearance in half-life. Hence, conclusively, as a result, more consideration should be given to the prescription and administration of *Angelica albicans* in colitis individuals, and more research is needed to determine whether the changed PK profile was clinically meaningful for medicinal dose.

## 1. Introduction

Angelica albicans is a valuable medicinal herb with a higher vitamin and mineral content that is widely utilized in food and supplements, as well as a natural herb [1]. Marsh tea with communal tansy was utilized as organic pest repellents. Angelica albicans has also been demonstrated to have antimicrobial, anti-inflammation, -asthma, -hypertensive, and -cancer activities in recent pharmacological research [2]. In longer-term clinical findings of colitis individuals, Angelica albicans alleviated the edema and atrophic patches of the colonic mucous membrane. Angelica albicans has been linked to the discovery of more than 70 coumarins. Furanocoumarins, such as imperatorin (IM) and isoimperatorin (ISOIM), are among the most active components of Angelica albicans [3]. They have a slew of biological features. IM and ISOIM have anticonvulsant, -hypertensive, vasodilator, -inflammatory, -spasmodic, and -cancer properties. The pharmacokinetics (PKs) of the two furanocoumarins in *Angelica albicans* must be assessed in a variety of disease conditions to provide more evidence about their effectiveness and also to truly comprehend the pharmacological underpinnings of their activities [4].

A recent study showed the adipogenic transcription factor peroxisome proliferator-activated receptor (PPAR), as well as the CCAAT enhancer-binding protein (C/EBP), was greatly boosted in mRNA and protein expression by ISOIM. Following isoimperatorin therapy, mRNA development of downriver adipogenesis-linked genomes sterol regulating element-binding transcription factor 1c, fatty-acid synthase, adiponectin, and so on, is grown dramatically. In 3 T3-L1 cells, ISOIM enhanced adipogenesis and vastly enhanced lipid formation in a dose-dependent way. ISOIM increased insulin signalling pathway stimulation by phosphorylating Akt, which is required for PPAR and C/EBP expression and transcription factors. Increased production of the genes FAS, DGAT2, and adiponectin, which are involved in 3 T3-L1 adipocyte development, may have resulted as a result of this [5]. Furthermore, through stimulating G protein-linked bile acid receptors 1 in mice, dietary furocoumarin IM, ISOIM isomer, promotes glucagon-based peptide production, lowering blood glucose [6]. Lipodystrophic individuals also have a triglyceride storage shortage in adipose tissue, which leads to ectopic lipid accumulation and severe insulin resistance [7, 8]. Furthermore, increased fatty capacity storage in adipose tissue paired with slower fat mobilization promotes fat mass growth may be the most efficient approach to store lipids in safe compartments [8]. In their study, they identified that the underlying mode of action through ISOIM which controls the diversity of 3 T3-L1 adipocytes is followed by the accretion of lipids. This research study can subsidize the growth of new medicines which can be used for the management of diabetes and other disorders.

*Angelica albicans* PK investigations in rats have been studied using a variety of analytical methodologies. Gas chromatography is used for measurement in rat plasma of IM [9]. Two ultrahigher-performance liquid methods based on mass spectroscopy (UPLC/MS/MS) by lesser quantification limits (LLOQ) of 5 ng/mL, run lengths of more than 20 minutes, or the need for big plasma samples were also factors. In rats, it was used to investigate the PKs of coumarins produced from herbal remedies [10].

Nonetheless, more sensitive, fast, and particular analytical techniques for instantaneous measurement of target analytes are still needed in PK research. As a result, a sensitivity UPLC/MS/MS technique for the detection of IM as well as ISOIM in rat plasma was developed and validated in this work. Functional and structural changes in the gastrointestinal system, such as lumen pH, flexibility, diarrhoea, and ulcer, can modify the PK profile of active substances administered orally [11]. We investigated whether the PK profiles of herbal therapies are altered in rats with experimentally-stimulated colitis because they are frequently administered orally.

The following is a summary of the research: Section 2 contains the methodology of the proposed work.  Section 3 discusses the experiment and results. Finally, the conclusion brings the paper to a finish in Section 4.

## 2. Methodology

**Figure 1:**
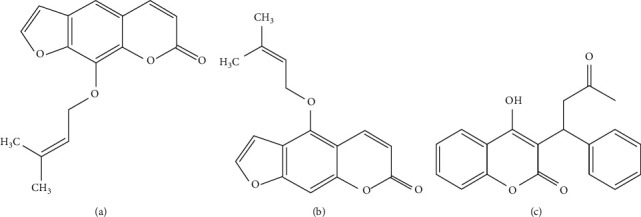
Chemical assemblies of (a) IM, (b) ISOIM, and (c) warfarin (internal standard: IS).

ChemFaces (Wuhan, China; Figure 1) provided IM and ISOIM. Sigma-Aldrich provided warfarin as a formic acid, IS, followed via 2,4,6-trinitrobenzene sulfonic acid (TNBS). “MS-grade” water and acetonitrile were used for MS research and plasma production. Raw *Angelica albicans* materials were attained. An ethanolic of *Angelica albicans*, a lyophilized brownish powder, was produced. *Angelica albicans* contained 6.67 and 2.34 mg/g extract of IM and ISOIM, respectively [12].

### 2.1. UPLC-MS/MS Study and Technique Corroboration

The *C*_max_ of two furanocoumarins is measured by employing a Thermo Q-Exactive equipped with an UltiMate 3000. A Hypersil GOLD column and a gradient method were used for the chromatography-based separation. By a 0.3 mL per minute flow rate, gradient elution is designed. Positive-ion mode, as well as a parallel reaction monitoring (PRM) approach, was used in the MS/MS study [13]. To obtain optimum sensitivity and selectivity with the PRM approach, the spray voltage, capillary temperature, sheath-, auxiliary-gaseous pressure, and resolution were set.  Table 1 shows the normalised collision energy for each analyte. Each plasma specimen was chemical degradation with acetonitrile containing IS, vortexed strongly for 5 minutes, and centrifuged to clean the plasma [14]. An aliquot of the filtered supernatant was then fed to the UPLC/MS/MS equipment. The linearity, selectivity, accuracy, precision, retrieval, matrix impacts, and durability of the approach model were all validated. For process corroboration, quality control specimens (*n* = 6) were used at 3 concentrations [15].

### 2.2. Animal Study

The China Animal Ethical centre approved for rats. Male Sprague-Dawley rats weighing 245–260 g were procured and acclimated for seven days in conventional laboratory settings by permitted access to food and water [15]. Thus, experimental colitis is produced by TNBS below isoflurane anaesthesia. The rats were starved for 24 hours before being infected with colitis. The rats are provided with one rectal dosage of TNBS over a pharmaceutical-grade polyurethane catheter in descending colon 8 cm from anus sphincter while under isoflurane anaesthesia [16]. To prevent intracolonic TNBS leaking, the rats are held in a supine Trendelenburg location for 3 minutes after gradually administering the TNBS over 1 minute. On day five of TNBS/50 percent ethanol therapy, normal rats were given 50 percent ethanol rather than TNBS, as well as the animals are employed in the PK investigation [17]. To prevent interfering with the colitis induction, no pain killers were given before the PK research. Normal and induced-colitis animals are randomly assigned to 1 of 4 groups: group 1, *Angelica albicans* 0.5 gram per kg in normal mice; group two, *Angelica albicans* 1 g per kg in normal rats; group three, *Angelica albicans* 0.5 gram per kg in stimulated-colitis mice; group four, *Angelica albicans* 1 g per kg in induced-colitis rats [18]. *Angelica albicans* was made with distilled water and given orally as a continuous gavage. Without anaesthesia, blood specimens are taken from the tail vein at regular intervals for 480 minutes. Blood specimens are centrifuged to extract the plasma, which was then kept at 80°C before usage.

### 2.3. Data Study

All of the data is given as a mean with standard deviation. The programme PK Solver was used to perform a noncompartmental PK study on the time concentration data of two furanocoumarins. Analysis of variance is utilized in comparison with PK constraints among normal and TNBS-treated rats. *p* values lesser than 0.05 are measured statistically important [19].

## 3. Results

### 3.1. Examination and Method Validation of UPLC/MS/MS

#### 3.1.1. Selectivity

**Figure 2:**
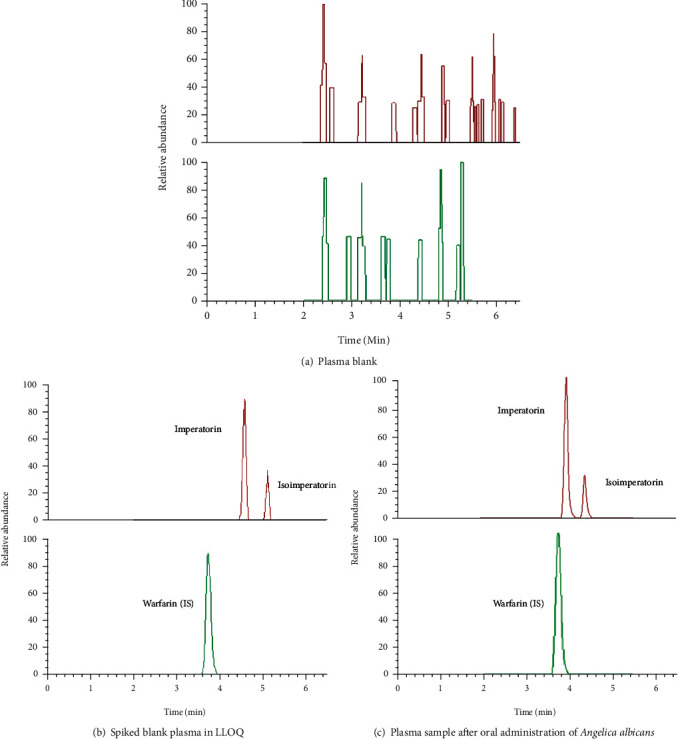
(a–c) PRM chromatogram of the analytes and plasma internal standard (IS).

**Figure 3:**
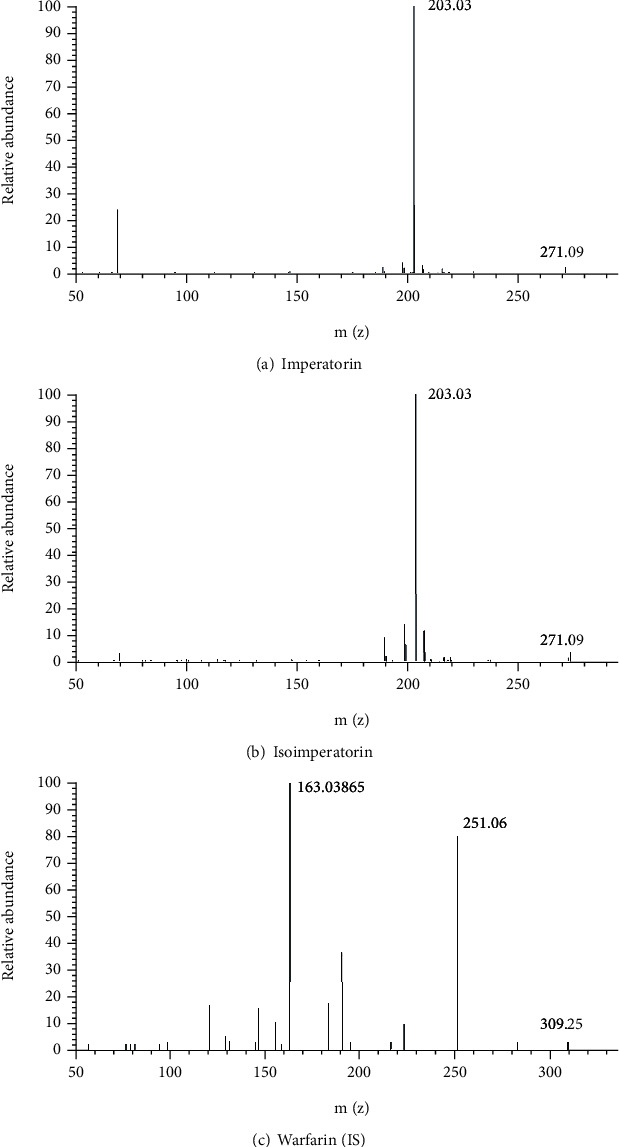
(a–c) Mass spectroscopy of the analytes and IS.

No endogenous intervention in peaking areas of IM at 4.51 min and ISOIM at 5.04 min or a standard solution in blank plasma with/without solutes and reliable plasma specimens underneath a founded UPLC/MS/MS analytical conditions [20] (Figure 2). The figure shows the 203.03345 from 287.09088, 203.03336 from 271.09573, 203.03336 from 271.09573, and most 163.03865 from 309.25220, respectively, were the most numerous and steady result ions of IM and ISOIM (Figure 3). Imperatorin, isoimperatorin, and warfarin (IS) were chosen from 287.09 to 203.03, 271.09 to 203.03, and 309.25 to 163.03.

Their standard curve was linear (*r* > 0.9994), so all analytes by the signal-to-noise ratio of 20 had a lower bound of quantitation (LLOQs) of 1.0 ng per mL (Table 2).

### 3.2. Accuracy

An intra- and interday correctness was reviewed in examinations of specimens for three concentrations of two furanocoumarins on the same day, three different days. All analytes on these day accurateness and precision were 6.9–6.8% and 1.3 percent to -9.4 percent (Table 3).

Table 4 shows an analytes removal efficiency, matrix impacts followed by stabilization in rat plasma. The target analytes were recovered at a rate ranging from 70.3 percent to 97 percent utilising liquid-liquid extraction. The removal efficiencies of acetonitrile, which was used for specimen preparation, were good, and all analytes were recovered reliably and consistently at all concentrations. There was no discernible matrix impact, and the matrix impacts ranged from 85% to 100%. During the specimen storage and processing methods, the target analytes' durability was assessed independently. All target solutes in rat plasma are durable below our simulated circumstances, with stability ranging from 94 percent to 101 percent.

The goal of this research was to create a simple, precise analytical approach for studying the PKs of IM and ISOIM. Due to a decline in blood volume caused by blood loss, the volume of blood serum specimens acquired and continued assembly in the body can modify PK parameters in PK experiments. In comparison to previously described approaches, this projected technique utilizes lesser plasma and required quicker run periods.

In comparison to the approach, the target analytes' sensitivities were increased by over fourfold. In conclusion, we devised a more sensitive, quick, and particular UPLC/MS/MS study, as well as a modest specimen preparation procedure, for the real-time measurement of two furanocoumarins in rats [21].

### 3.3. Uses

#### 3.3.1. Induction of Experimental Colitis

**Figure 4:**
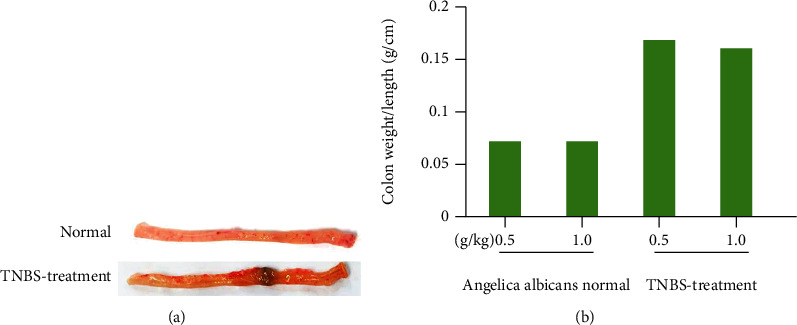
TNBS-stimulated experiment-based colitis in rats. (a) Macroscopic presence of normal and injured colon and (b) mass of explanted colon.

Within three to seven days after rectal treatment of TNBS, rats develop acute inflammatory and substantial disruption to an intestinal barricade [22]. Ethanol breaks down the mucosa barrier, and TNBS haptenizes autologous colonies as well as bacterial receptors via the human immune system. The induced-colitis rats in groups 3 and 4 had bloody stools and lost 12.5% and 13.2% of their body weight, respectively. TNBS induced severe ulceration at the instillation area as well as a considerable rise in colon weight, according to necropsy Figure 4. TNBS-treated rats were utilized to see how colitis affects the PKs of furanocoumarins in *Angelica albicans*.

#### 3.3.2. PKs of Angelica albicans in Untreated and Colitis-Treated Rats

**Figure 5:**
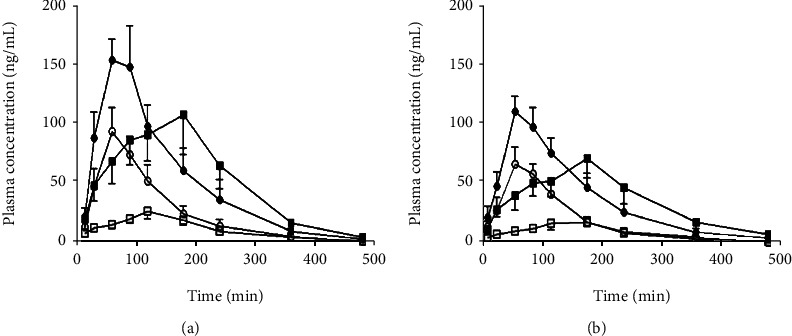
*C*_max_–time curves of (a) IM and (b) ISOIM after oral administration of Angelica albicans.

After orally administered *Angelica albicans*, Figure 5 depicts the mean *C*_max_–time curves of IM and ISOIM, and Table 5 recapitulates their important PK properties. The *T*_max_ of the two furanocoumarins varied depending on the dose of *Angelica albicans* delivered (0.5 or 1 gram per kg) in both normal and TNBS-used mice [23]. Oral IM and ISOIM PK investigations in normal, as well as treated-colitis rats (*n* = 6), were effective, and two furanocoumarins are verified as biologically available active ingredients of *Angelica albicans*.

All two furanocoumarins reached maximum plasma levels in normal rats between 40 and 75 minutes, indicating rapid oral absorption. The periods to attain maximal concentration (*T*_max_) of the two furanocoumarins, on the other hand, were shorter than in earlier studies. The PK profile of one component and the *Angelica albicans* extract after oral administration may differ due to administration composition. In this investigation, the *Angelica albicans* dosages are 4.5 and 9.0 times lesser than those utilized in another study. Extra dosages have been demonstrated to impact PK parameters during the absorption phases in PK studies. The variation in *T*_max_, in this case, could be attributable to variances in the quantity of furanocoumarin as well as nonfuranocoumarin constituents are the consequence of an *Angelica albicans* manufacturing process and the test substance dose. The *T*_max_ of IM and ISOIM was significantly delayed in colitis rats, ranging from 113 to 144 minutes. The *C*_max_ of IM and ISOIM dropped dramatically to around 50% after *Angelica albicans* injection. The MRT of IM and ISOIM, on the other hand, was increased by 40% to 65% (p 0.05). Comparing normal and colitis-induced rats, the area underneath the *C*_max_–time curve, as well as the exclusion *t*_1/2_ of the two furanocoumarins, are not substantially diverse. The delayed *T*_max_ and lowered *C*_max_ in colitis-induced rats could be affected during the medication absorption period.

## 4. Conclusion

In PK rat research, a quick, sensitive UPLC/MS/MS technique for its purpose of two furanocoumarins was identified and utilized to investigate the impact of colitis. The effects of colitis on the pace and degree of oral absorption of key bioactive components following *Angelica albicans* administration were initially observed; however, the processes are unknown. As a result, more consideration should be given to the prescription and administration of *Angelica albicans* in colitis individuals, and more research is needed to determine whether the changed PK profile was clinically meaningful for medicinal dose. Modifications in physiological milieu associated with gastrointestinal tract illnesses, such as colitis, might impact intestinal absorption when therapeutic drugs are administered orally, resulting in therapeutic failure or harmful consequences. Dyspepsia, intestinal hypomotility, and late gastric emptying were all symptoms of colitis, as are structural alterations caused by inflammation infiltrates, tissue edema, and ulceration. Dyspeptic symptoms and gastroparesis, which result in lessened and deferred stomach emptying, are common in colitis individuals, and late gastric emptying can affect PK characteristics of oral medications including *T*_max_ and *C*_max_. In rats with TNBS-induced colitis, a neurological pathway connecting pelvic afferent nerve hyperactivity prevented stomach emptying from causing an increase in intestinal transit. Furthermore, colitis-induced changes in CYP expression and decreased metabolic activity may have an impact on the plasma levels of CYP-metabolized medicines. A study discovered that in TNBS-treated rats, the levels and activity are reduced. IM and ISOIM are converted by liver microsomes into xanthotoxol and heraclenin, through demethylation, oxidation, and so on, and these metabolites have been found in rat plasma, bile, and urine. Without AUC_0 ∞_, TNBS-treated colitis caused a reduction in *C*_max_ as well as an interruption in *T*_max_, suggesting reduced oral absorption of the two furanocoumarins in the current investigation. In pathophysiological settings, an additional mode of study of aspects governing their oral absorption, such as below the conditions indicated above, was required.

## Figures and Tables

**Table 1:** The concept and data on PRM features.

Components	Formula	Precursor ion [M + H]^+^		Synthesis	Normalized collision energy
		Estimated	Measured		
IM	C_16_H_14_O_4_	281.096	281.095	213.033	20
ISOIM	C_16_H_14_O_4_	281.096	281.096	213.033	20
Warfarin (IS)	C_19_H_16_O_4_	319.112	319.252	261.069	50

**Table 2:** Calibration and linearity curves of IM and ISOIM.

Constituent	Range (ng/ML)	Equation of linear regression	Correlation coefficient	LLOQ (ng/mL)
IM	1-200	*Y* = 0.08*x* + 0.013	0.99	1
ISOIM	1-200	*Y* = 0.04*x* + 0.025	0.99	1

**Table 3:** Precision and accuracy of IM and ISOIM in rat plasma.

Constituents	Conc. nominal (ng/mL)	Intraday			Interday		
		Estimated conc. (ng/mL)	RE (%)	RSD (%)	Estimated conc. (ng/mL)	RE (%)	RSD (%)
IM	2	1.90	-6.90	8.30	1.90	-4.90	8.70
	50	49.40	-2.20	3.90	49.90	-0.50	4.50
	150	149.10	-0.60	3.80	151.70	1.20	3.10
ISOIM	2	1.90	-5.10	07	02	-4.10	4.40
	50	50.30	01	3.60	48.60	-5.60	2.80
	150	150.20	0.10	1.90	151.40	0.90	1.80

**Table 4:** Extraction recovery, matrix impacts, and stability of IM and ISOIM in rat plasma.

Constituents	Conc. nominal (ng/mL)	Recovery rate in %	Matrix (%)	Durability (%)		
				Free-thaw cycles	At -70 °C for 30 days	At room *T* for 24 hrs
IM	2	84.9	91	97	99	97
	50	83.4	92	100	101	100
	150	79.8	85	101	94	101
ISOIM	2	85.3	94	101	98	95
	50	75.6	96	98	98	100
	150	70.3	94	95	97	97

**Table 5:** Pharmacokinetic properties of IM and ISOIM after oral administration of *Angelica albicans* to normal and experimental-based TNBS-treated rat.

Properties	Normal rat		TNBS-treated rat	
	0.5 g/kg	1 g/kg	0.5 g/kg	1 g/kg
IM	59	61	65	56
*t*_1/2_ (min)	54	72	113	127
*T*_max_ (min)	94	201	27	118
*C*_max_ (ng/mL)	11860	23889	5196	24845
AUC_0_ → _∞_ MRT (min)	121	127	172	187
ISOIM	63	78	88	68
*t*_1/2_ (min)	72	67	120	144
*T*_max_ (min)	72	128	21	71
*C*_max_ (ng/mL)	9445	17778	4197	17881
AUC_0_ → _∞_ MRT (min)	143	145	204	225

*t*_1/2_ denotes half-life, *T*_max_ represents time to attain peak concentration, *C*_max_ signifies plasma concentration peak, →AUC_0 ∞_ is the area in plasma concentration, MRT means mean residence time, ∗*p* < 0.05 represents comparison of dosage treated normal rats.

## Data Availability

The data used to support the findings of this study can be obtained from the corresponding author upon reasonable request.
